# Acerola (*Malpighia emarginata DC*.) juice intake protects against alterations to proteins involved in inflammatory and lipolysis pathways in the adipose tissue of obese mice fed a cafeteria diet

**DOI:** 10.1186/1476-511X-13-24

**Published:** 2014-02-04

**Authors:** Fernando Milanez Dias, Daniela Dimer Leffa, Francine Daumann, Schérolin de Oliveira Marques, Thais F Luciano, Jonathan Correa Possato, Aline Alves de Santana, Rodrigo Xavier Neves, José Cesar Rosa, Lila Missae Oyama, Bruno Rodrigues, Vanessa Moraes de Andrade, Cláudio Teodoro de Souza, Fabio Santos de Lira

**Affiliations:** 1 Laboratory of Molecular and Cellular Biology, Graduate Programme of Health Sciences, Department of Health Sciences, Universidade do Extremo Sul Catarinense, UNESC, 888.06-000 Criciúma, SC, Brazil; 2 Laboratory of Exercise Biochemistry and Physiology, Graduate Programme of Health Sciences, Department of Health Sciences, Universidade do Extremo Sul Catarinense, UNESC, 888.06-000 Criciúma, SC, Brazil; 3 Departamento de Fisiologia, Disciplina de Fisiologia da Nutrição, Universidade Federal de São Paulo (UNIFESP), 04023-060 São Paulo, SP, Brazil; 4 Cancer Metabolism Research Group, Institute of Biomedical Sciences, University of Sao Paulo (USP), São Paulo, Brazil; 5 Immunometabolism Research Group, Institute of Biomedical Sciences, University of São Paulo (USP), São Paulo, Brazil; 6 Laboratório do Movimento Humano, Universidade São Judas Tadeu, São Paulo, SP, Brazil; 7 Immunometabolism Research Group, Department of Physical Education, Universidade Estadual Paulista, UNESP, Rua Roberto Simonsen, 305, 19060-900 Presidente Prudente, SP, Brazil

**Keywords:** Cafeteria diet, *Malpighia emarginata* DC, Adipose tissue, Inflammation, Lipolysis

## Abstract

**Background:**

Obesity has been studied as a metabolic and an inflammatory disease and is characterized by increases in the production of pro-inflammatory adipokines in the adipose tissue.

To elucidate the effects of natural dietary components on the inflammatory and metabolic consequences of obesity, we examined the effects of unripe, ripe and industrial acerola juice (Malpighia emarginata DC.) on the relevant inflammatory and lipolysis proteins in the adipose tissue of mice with cafeteria diet-induced obesity.

**Materials/methods:**

Two groups of male Swiss mice were fed on a standard diet (STA) or a cafeteria diet (CAF) for 13 weeks. Afterwards, the CAF-fed animals were divided into five subgroups, each of which received a different supplement for one further month (water, unripe acerola juice, ripe acerola juice, industrial acerola juice, or vitamin C) by gavage. Enzyme-linked immunosorbent assays, Western blotting, a colorimetric method and histology were utilized to assess the observed data.

**Results:**

The CAF water (control obese) group showed a significant increase in their adiposity indices and triacylglycerol levels, in addition to a reduced IL-10/TNF-α ratio in the adipose tissue, compared with the control lean group. In contrast, acerola juice and Vitamin C intake ameliorated the weight gain, reducing the TAG levels and increasing the IL-10/TNF-α ratio in adipose tissue. In addition, acerola juice intake led to reductions both in the level of phosphorylated JNK and to increases in the phosphorylation of IκBα and HSL^ser660^ in adipose tissue.

**Conclusions:**

Taken together, these results suggest that acerola juice reduces low-grade inflammation and ameliorates obesity-associated defects in the lipolytic processes.

## Introduction

Obesity is characterized by an excessive accumulation of body fat due to a chronic state of positive energy balance resulting from unhealthy dietary habits and sedentary physical activity patterns [1]. Adipose tissue plasticity is coordinated through several steps, and increases in the fatty mass come about through an unbalance between lipolytic processes, which involve two principal lipases, hormone sensitive lipase (HSL) and adipose triglyceride lipase (ATGL) [2], and lipogenesis processes, which are mainly controlled by fatty acid synthase (FAS) and acetyl CoA carboxylase (ACC) [3].

Obesity has been studied as a metabolic and an inflammatory disease and is characterized by increases in the production of pro-inflammatory adipokines in the adipose tissue, which establish systemic low-grade inflammation [4]. Obesity has also become one of the foremost problems facing public health; its incidence is associated with an elevated risk for many types of cancer, cardiovascular diseases, dementia, type 2 diabetes and other co-morbidities.

Along with the increasing prevalence of obesity, a number of drugs have been developed to treat the associated metabolic disruptions, focusing on achieving increased fat mobilization and oxidation and decreased fat absorption and appetite [5]. However, these drugs have generally been unsuccessful due to their low efficacy or large side effects [6].

In recent years, numerous bioactive compounds in fruits have been explored for their potential anti-obesity effects; the nutritional components of fruits have the potential to enact a number of positive health effects toward the prevention of lifestyle-related diseases [6,7]. Acerola (*Malpighia emaginata* DC.) is a fruit found throughout Central America and into the northern part of South America. This fruit is well known as one of the best natural sources of vitamin C and has become extremely popular among the health-conscious population [8]. Aside from vitamin C, acerola contains other similarly functional ingredients, such as carotenoids, gamma-amino butyric acid (GABA) and polyphenols [9].

Dietary bioactive compounds have been demonstrated to possess anti-inflammatory effects on the adipose tissue by several mechanisms [1]. Previous studies [10] and [11] have shown that the nuclear factor kappa B (NFκB) transcription factor is a key mediator of inflammation in adipocyte cells. Currently, studies have shown a close relationship between toll-like receptor 4 (TLR-4) and the activation of the NF-κB pathway, which leads to the elevation of pro-inflammatory adipokine genes and protein expression in adipose tissues [11,12]. In addition, Feingold et al. [13] demonstrated that pro-inflammatory adipokines induce lipolysis, principally, IL-6 and TNF-α. However, the opposite is also true; free fatty acid can also lead to inflammation [14]. In 2005, Suganami et al. [15] showed that free fatty acids produced by lipolysis in adipocytes activated monocytes, creating a paracrine loop between lipolysis and inflammation. However, at present, we understand that adipocytes express several members of the family of Toll-Like Receptors (TLRs), which can recognize free fatty acids and induce the production of pro-inflammatory adipokines by adipocytes [6].

However, relatively little is known about the underlying mechanisms regulating body weight, lipolytic actions and the relevant inflammatory status. Thus, the aims of this study were to examine the effects of acerola juice on body fat mass and the presence of lipolytic enzymes in adipose tissue in mice fed a cafeteria diet and to observe whether reducing the fat mass is associated with a diminished level of low-grade inflammation.

## Methods and materials

### Animals

Thirty-six male Swiss albino mice, at 25 grams of body weight and 5–6 weeks of age, were obtained from the Animal Center from Universidade do Extremo Sul Catarinense (UNESC, Brazil). The experimental protocols for this study were approved by the Local Ethics Committee for Animal Use (CEUA) of UNESC, Brazil (register No. 130/2011). The mice were randomized by weight and housed at 6 animals per cage under standard room temperature (22 ± 2°C) under a 12 h light/dark cycle. The animals were acclimated to their environment for 1 week before the beginning of the experiment. Thirty-six animals were divided into 2 groups, a control group (6 animals) and cafeteria diet group (30 animals). The animals were fed with a standard diet (STA) or cafeteria diet (CAF) for the 13 weeks. After this period, the animals of the CAF group were subdivided into five subgroups (6 animals/group): mice treated with vehicle (CAF + water distilled), mice treated with 1 mg/kg of vitamin C (CAF + Vitamin C), and mice treated with 0.1 mL/10 g of animal per day with three different acerola juices: industrial (CAF + Industrial), unripe (CAF + Unripe) and ripe (CAF + Ripe). The industrial acerola juice was obtained from Da Fruta® (Pernambuco, Brazil). The unripe and ripe acerola (*Malpighia emerginata* DC.) juices were purchased from the Nutrilite Farm (Ceará, Brazil). L-ascorbic acid (Chemical Abstract Service Register Number 50-81-7) was purchased from Sigma–Aldrich (Porto Alegre, Brazil), and to obtain the desired final dose, vitamin C was dissolved in distilled water just before each experiment. All of these diets were administered to the animals by gavage. The animals were weighed weekly, and their food intake was recorded daily (*data not shown*). After 4 weeks on these different treatments, the animals of each group were sacrificed. Then, blood samples were collected to evaluate the TAG levels, and epididymal adipose tissue pads were dissected for histology, immunoassay, and western blot analysis. All adipose tissues (epididymal, mesenteric, and retroperitoneal) were weighed for adiposity index.

### Experimental diets

The STA (Nuvilab CR-1, NUVITAL®, Curitiba, PR, Brazil) provided an energy content of 2.93 kcal/g, and the CAF totaled 4.12 kcal/g (the constituents of each diet are described in Table 1). The palatable high-calorie diet (cafeteria diet) was chosen because mimics the modern patterns of human food consumption and has been usefully in other experimental studies to induce obesity in lean animals. This diet was adapted from a diet known as the cafeteria diet or Western diet, previously described by Shafat et al. [16]. Both the standard chow and the experimental diet were replaced daily with fresh food. The animals receiving the cafeteria (hypercaloric diet) and standard diet had free access to standard chow and water (see weekly menu in Table 2).

**Table 1:** Macronutrient composition of the diets

**Composition**	**Standard diet**		**Cafeteria diet**	
	**(2.93 Kcal/g)**		**(4.12 Kcal/g)**	
	**g/100 g**	**Kcal/100 g**	**g/100 g**	**Kcal/100 g**
Protein	22.00	88.00	7.67	30.68
Carbohydrate	53.00	212.00	41.97	167.88
Saturated fat	4.00	36.00	23.76	213.84

**Table 2:** Weekly menu of animals fed with cafeteria diet

**Days of week**	**Food**
Monday	Mortadella, marshmallow, cheese chips, chocolate wafer, chow *Nuvilab*®, water and *Guaraná* soft drink
Tuesday	Chocolate crackers, Doritos®, chow *Nuvilab*®, sausage hot dog, water and *Guaraná* soft drink
Wednesday	*Paçoca* peanuts, chow *Nuvilab*®, mortadella, cheese chips, water and *Guaraná* soft drink
Thursday	Mortadella, chocolate wafer, calf's foot jelly, Doritos®, chow *Nuvilab*®, water and cola soft drink
Friday, Saturday and Sunday	Chocolate crackers, chow *Nuvilab*®, sausage hot dog, bacon chips*,* marshmallow*,* water and cola soft drink

### Serum triacylglycerol assay

Fasting animal serum triglyceride levels were assessed by the colorimetric method with a commercial kit Labtest (Brazil).

### TNF-α, and IL-10 protein level determination by ELISA

Following decapitation, epididymal adipose tissue was removed, dissected, homogenized, and centrifuged at 12,000 g for 40 min at 4°C. The supernatants were used for protein quantification, according to the Bradford method, with bovine serum albumin (BSA) as a reference. The quantitative assessment of TNF-α and IL-10 proteins was carried out by ELISA (DuoSet ELISA, R & D Systems, Minneapolis, MN), following the recommendations of the manufacturer. All samples were run as duplicates, and the mean value is reported.

### Protein analysis by immunoblotting

Epididymal adipose tissue was homogenized immediately in extraction buffer (1% Triton-X 100, 100 mM Tris, pH 7.4, containing 100 mM sodium pyrophosphate, 100 mM sodium fluoride, 10 mM EDTA, 10 mM sodium vanadate, 2 mM PMSF and 0.1 mg of aprotinin/ml) at 4°C (Polytron MR 2100, Kinematica, Switzerland). The extracts were centrifuged at 9,000 × g and 4°C (5804R, Eppendorf AG, Hamburg, Germany) for 40 min to remove insoluble materials. The supernatants were used for protein quantification, according to the Bradford method. The proteins were denatured by boiling in Laemmli sample buffer containing 100 mM DTT, run on SDS-PAGE, and transferred to nitrocellulose membranes. The membranes were blocked, probed and blotted with primary antibodies. The antibodies used for immunoblotting were phospho AMPK^Thr172^, phospho HSL^serine563^, phospho HSL^serine660^ (Cell Signaling Biotechnology, Beverly, MA, USA) and perilipina A, CGI-58, ATGL, phospho IκB-α^serine32/36^, phospho JNK^183/Tyr185^, NF-κBp50, and TLR4 (Santa Cruz Biotechnology, Santa Cruz, CA, USA). The original membrane was stripped and reblotted with α-tubulin loading protein. Chemiluminescent detection was performed with horseradish peroxidase-conjugate secondary antibodies (Thermo Scientific, Rockford, IL, USA). Autoradiographs of membranes were taken for the visualization of protein bands. The results of the blots are presented as direct comparisons of the area of apparent bands in autoradiographs and were quantified by densitometry using the Scion Image software (Scion Image software, ScionCorp, Frederick, MD).

### Histological technique

Epididymal adipose tissue samples were prepared in paraffin blocks, which subsequently were subjected to 4 μm-thick histological sectioning for slide preparation, always stained with hematoxylin-eosin. All slides were examined under an optical microscope by a pathologist who was unaware of the origin of the material and of the objectives of the study. Using a program for image analysis, the computer-assisted Image-Pro Plus 6.0 (Media Cybernetics, Bethesda, MD, USA), we were able to measure the area, mean diameter and perimeter.

### Statistical analysis

The data were expressed as means ± SEM. The normality of the variables was evaluated using the Kolmogorov–Smirnov test. First, the unpaired t-test was used to evaluate the standard and cafeteria diets. Next, the different treatments (water, unripe acerola juice, ripe acerola juice, industrial acerola juice, and vitamin C) were analyzed using one-way analysis of variance (ANOVA). When ANOVA showed significant differences, post hoc analysis was performed with the Tukey’s test. A probability of less than 0.05 was considered significant. The software used to analyze the data was the Statistical Package for the Social Sciences (SPSS) version 16.0 for Windows.

## Results

**Figure 1:**
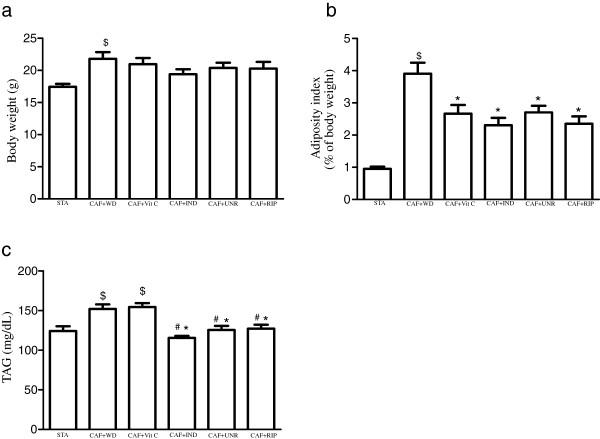
**Effects of cafeteria diet on metabolic parameters.** Analysis of body weight (g) **(a)**; adiposity index (% of body weight) **(b)**; and circulating TAG (mg/dL) **(c)** of mice fed the standard diet (STA), the cafeteria diet with distilled water (CAF + WD), the cafeteria diet with vitamin C (CAF + Vit C), the cafeteria diet with industrial acerola juice (CAF + IND), the cafeteria diet with the juice of unripe acerolas (CAF + UNR) or the cafeteria diet with the juice of ripe acerolas (CAF + RIP). The results are expressed as means ± SEM (n = 6 per group). ^$^p < 0.05 versus standard diet; *p < 0.05 versus the cafeteria diet with distilled water; ^#^p < 0.05 versus the cafeteria diet with vitamin C.

First, we evaluated the effects of the cafeteria diet on the body weight of the mice. The cafeteria diet increased the body weight in the CAF + WD group when compared with standard diet group (p < 0.01). Although the treatment not was efficient in reducing the weight of the experimental mice (p > 0.05; Figure 1a), an interesting result was observed in the adiposity index. The cafeteria diet markedly increased the adiposity index in the CAF + WD group when compared with STA group (p < 0.05), while the CAF + Vit C, CAF + IND, CAF + UNR, and CAF + RIP groups exhibited a reduced adiposity index when compared with the CAF + WD group (p < 0.05; Figure 1b). No significant difference was found among the treated groups (p > 0.05). To determine the adiposity index, the epididymal, mesenteric and retroperitoneal pads were weighed. To observe whether the treatment induced increased lipolysis, we assayed the triacylglycerol levels. The TAG levels were increased in the CAF + WD and CAF + Vit C groups when compared with the STA group (p < 0.05). However, the TAG levels in the CAF + IND, CAF + UNR, and CAF + RIP groups were reduced when compared with the CAF + WD and CAF + Vit C groups (p < 0.05; Figure 1c).

**Figure 2:**
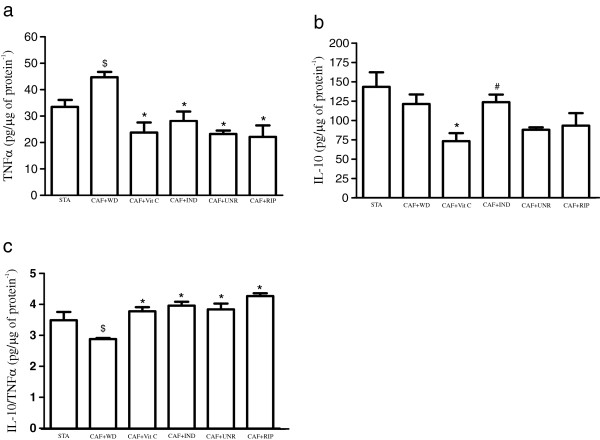
**Effects of cafeteria diet on inflammatory parameters in epididymal adipose tissue.** TNF-α (pg/μg protein^-1^) **(a)**; IL-10 (pg/ug protein^-1^) **(b)**; and IL-10/TNF-α ratio **(c)** of mice fed the standard diet (STA), the cafeteria diet with distilled water (CAF + WD), the cafeteria diet with vitamin C (CAF + Vit C), the cafeteria diet with industrial acerola juice (CAF + IND), the cafeteria diet with the juice of unripe acerolas (CAF + UNR) or the cafeteria diet with the juice of ripe acerolas (CAF + RIP). The results are expressed as means ± SEM (n = 6 per group). ^$^p < 0.05 versus standard diet; *p < 0.05 versus the cafeteria diet with distilled water; ^#^p < 0.05 versus the cafeteria diet with vitamin C.

We observed that the TNF-α levels were increased in the CAF + WD group when compared with the STA group (p < 0.05), although the TNF-α levels were reduced in all of the treated groups when compared with the CAF + WD group (p < 0.05). There was no difference in the TNF-α levels among the treatment groups (p > 0.05; Figure 2a). When the IL-10 levels (an important anti-inflammatory cytokine) were analyzed, we observed that the IL-10 levels were unchanged between the CAF + WD and STA groups (p > 0.05), even though the IL-10 levels were reduced in the CAF + Vit C group when compared with the CAF + WD group (p < 0.05). In addition, the IL-10 levels were higher in the CAF + IND group than in the CAF + Vit C group (p < 0.05; Figure 2b). We observed that the IL-10/TNF-α ratio was reduced in the CAF + WD group when compared with the standard diet group (p < 0.05). However, the IL-10/TNF-α ratio in the CAF + Vit C, CAF + IND, CAF + UNR, and CAF + RIP groups was increased when compared with the CAF + WD group (p < 0.05; Figure 2c).

**Figure 3:**
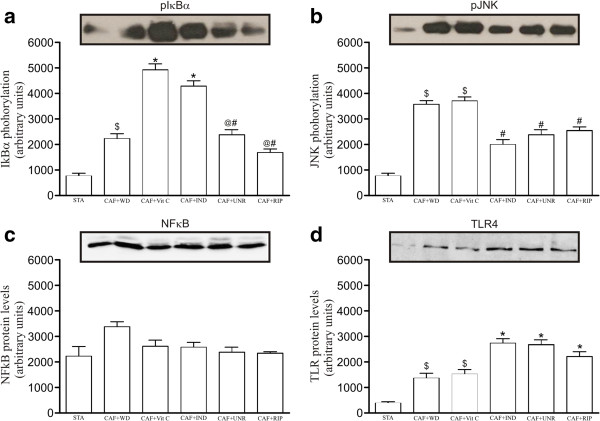
**Effects of cafeteria diet on inflammatory molecules in epididymal adipose tissue.** The levels of phosphorylated pIκB **(a)**; pJNK **(b)**; NF-κB **(c)**; and TLR4 **(d)** in the adipose tissue of mice fed the standard diet (STA), the cafeteria diet with distilled water (CAF + WD), the cafeteria diet with vitamin C (CAF + Vit C), the cafeteria diet with industrial acerola juice (CAF + IND), the cafeteria diet with the juice of unripe acerolas (CAF + UNR) or the cafeteria diet with the juice of ripe acerola (CAF + RIP). The results are expressed as means ± SEM (n = 6 per group). ^$^p < 0.05 versus standard diet; *p < 0.05 versus the cafeteria diet with distilled water; ^#^p < 0.05 versus the cafeteria diet with vitamin C; ^@^p < 0.05 versus the cafeteria diet with industrial acerola juice.

**Figure 4:**
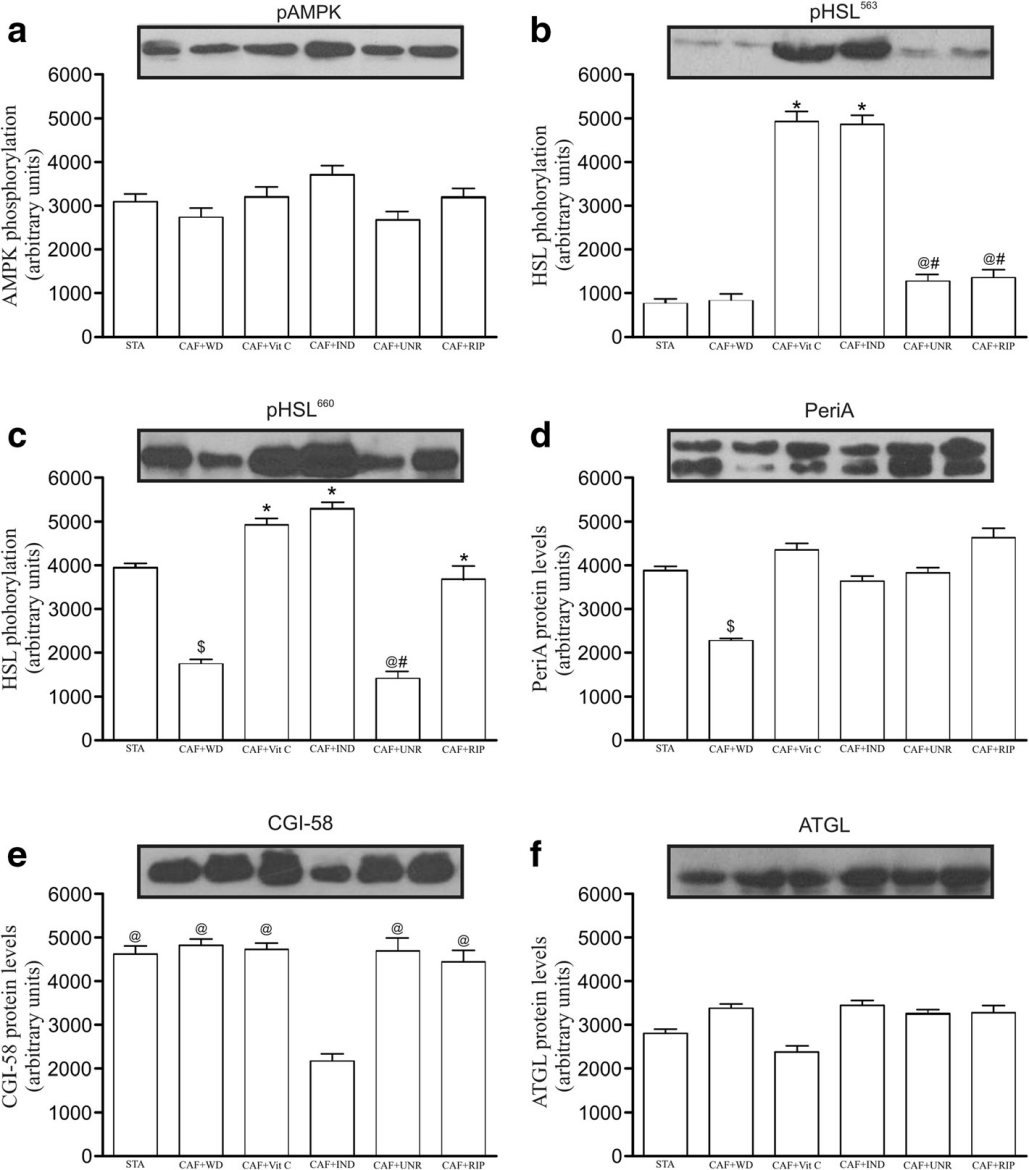
**Effects of cafeteria diet on lipolysis molecules in epididymal adipose tissue*.*** The levels of phosphorylated AMPK^Thr172^**(a)**; HSL^Ser563^**(b)**; HSL^Ser660^**(c)**;and protein levels the Peri A **(d)**; CGI-58 **(e)**; and ATGL (**f**) in the adipose tissue of mice fed the standard diet (STA), the cafeteria diet with distilled water (CAF + WD), the cafeteria diet with vitamin C (CAF + Vit C), the cafeteria diet with industrial acerola juice (CAF + IND), the cafeteria diet with the juice of unripe acerolas (CAF + UNR) or the cafeteria diet with the juice of ripe acerola (CAF + RIP). The results are expressed as means ± SEM (n = 6 per group). ^$^p < 0.05 versus standard diet; *p < 0.05 versus the cafeteria diet with distilled water; ^#^p < 0.05 versus the cafeteria diet with vitamin C; ^@^p < 0.05 versus the cafeteria diet with industrial acerola juice.

Next, we evaluated the protein levels of the molecules involved in the transduction of pro-inflammatory signals (pIκ-Bα, pJNK, NF-κB, TLR4), which are presented as representative bands. The pJNK protein levels were reduced in the CAF + IND, CAF + UNR, and CAF + RIP groups (p < 0.05; Figure 3b) when compared with the CAF + WD group. There was no difference among the groups in the NF-κBp50 protein levels (p > 0.05; Figure 3c). Hoping to evaluate whether lipolytic enzymes were altered by the treatment, we evaluated the levels of pAMPK, pHSL^ser563^, pHSL^ser660^, PeriA, CGI-58, and ATGL. The results demonstrated a higher phosphorylation of HSL at the serine 563 and 660 sites in the CAF + Vit C and CAF + IND groups (p < 0.05) when compared with the CAF + WD group, suggesting increased lipolytic activity in these groups (Figure 4).

**Figure 5:**
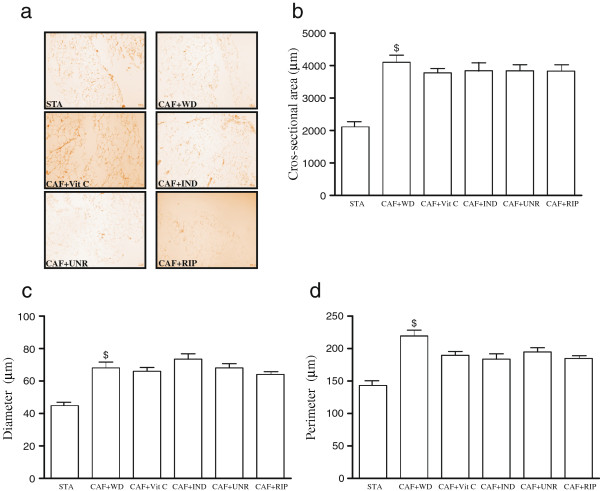
**Effects of cafeteria diet on histological-morphometrical in epididymal adipose tissue.** Images of hematoxylin and eosin **(a)**; cros-sectional area (μm) **(b)**; diameter (μm) **(c)**; perimeter (μm) **(d)** in the adipose tissue of mice fed the standard diet (STA), the cafeteria diet with distilled water (CAF + WD), the cafeteria diet with vitamin C (CAF + Vit C), the cafeteria diet with industrial acerola juice (CAF + IND), the cafeteria diet with the juice of unripe acerolas (CAF + UNR) or the cafeteria diet with the juice of ripe acerola (CAF + RIP). The results are expressed as means ± SEM (n = 6 per group). ^$^p < 0.05 versus standard diet.

In order to identify size of adipocytes, hematoxylin and eosin was performed (Figure 5). The results show that all cafeteria diet groups exhibited increased area, diameter and perimeter when compared with standard diet.

## Discussion

In the present study, we showed that the intake of acerola juice decreased the level of inflammatory proteins (TNF-α) and increased lipolysis in mice fed a cafeteria diet.

The adipose tissue metabolism and plasticity are still complex topics. Numerous lipolytic and antilipolytic effectors, including hormones, cytokines, and adipokines, control the catabolism of stored fat in various tissues. Since 1992, the link between inflammation and lipolysis has been studied in regard to pro-inflammatory adipokine-induced lipolysis, principally focusing on IL-6 and TNF-α [13]. We evaluated the phosphorylation levels of two serine residues, HSL (563 and 660), which we regarded as acting on β-adrenergic receptors. We observed that principally the phosphorylation of HSL^Ser660^ and Perilipin A protein levels were reduced in the adipose tissues of mice who were submitted to a cafeteria diet, while mice receiving acerola juice had increased levels of phosphorylated HSL^Ser660^ and Perilipin A. The results may be at least partially responsible for the minor adiposity index after treatment with acerola juice, in comparison with mice fed only a cafeteria diet.

Studies have shown that increased body fat can lead to low-grade inflammation (i.e., increased cytokines). As we observed reductions in the adiposity index, we hypothesized that the cytokine levels may also be reduced. With this in mind, we assayed the levels of TNF-α and IL-10 and calculated the IL-10/TNF-α ratio. We observed the increased phosphorylation of IκB-α, which can be explained by the reduction in TNF-α protein levels. This increase in the IL-10/TNF-α ratio and protein levels in the adipose tissue was observed in mice treated with acerola juice. Thus, the IkB-α levels must reflect the activity of the transcription factor NF-κB [17].

Our results showed that both acerola juice (industrial, unripe, ripe) and Vitamin C treatment lead to improved metabolic and inflammatory pathways. The effects of acerola juice observed in present study can be attributed to the polyphenol content, Vitamin C, quercetin and rutin [9].

Several studies have shown that high-fat diet-induced adiposity can be reduced by Vitamin C supplementation in rats [18,19] and by modifications to adipocyte catecholamine-induced lipolysis [20]. In addition, Garcia-Diaz et al. [21] demonstrated that Vitamin C supplementation can modulate an established inflammatory state in the interaction between adipocytes and macrophages. Moreover, resident macrophages produce catecholamines, which stimulate lipolysis in white adipose tissue [22]. This pathway may be implicated in the central mechanisms of the increased lipolysis and anti-inflammatory effects of acerola juice, because IL-4, an anti-inflammatory cytokine, is necessary for the activation of lipolysis. The associated increase in catecholamine production in macrophages [22] may explain the increased level of IκB-α proteins and reduction in TNF-α protein levels.

However, others biocompounds exist in acerola juice that may be associated with the observed effects on metabolism and inflammation. Rivera et al. [23] related that the administration of quercetin reduced insulin resistance, dyslipidemia, and hypertension in rats. More recently, Overman et al. [24] reported that quercetin reduces inflammation in adipose tissue by lowering the infiltration of macrophages and suppressing NF-κB activation. We observed an increased level of phosphorylated Iκ-Bα proteins, which may indicate a suppression of NF-κB activation, confirming the observations of Overman and colleagues [24].

Hsu et al. [25] showed that the body, liver organ, and adipose tissue weights of the peritoneal and epididymal fat pads of mice on a high fat diet supplemented with rutin were significantly decreased, compared with those on a high fat diet without supplementation. In addition, the authors verified that the serum lipid profiles, insulin, and leptin were significantly decreased after treatment with rutin. Recently, Gao et al. [26] showed that rutin is able to block high fat diet-induced obesity, fatty liver and insulin resistance in mice. Further, these beneficial effects were correlated with a blockade of macrophage infiltration and chronic inflammation in the adipose tissues. In our study, we observed reduced TAG levels in accordance with the above cited studies.

Acerola juice suppresses glucose absorption and blood glucose elevation after feeding [9]. This decrease in glucose disposition by the energetic metabolism can be responsible, due to the increased fatty acid utilization, for maintaining the energetic demand, ameliorating the vicious cycle of lipolysis-inflammation, and mobilizing the free fatty acids from lipolysis to be used as an energy source. However, recently, Leffa et al. [27] showed that acerola juice is not able to alter or reverse insulin resistance in mice fed a high-fat diet. More studies are needed to achieve a better understanding of the involved mechanisms.

Finally, to verify the area of the adipocytes, we evaluated the histology with hematoxylin and eosin (HE). The results demonstrated, at least in part, that all cafeteria diet groups had an increased area of adipocytes when compared with the STA group, regardless of the effects of the different treatments.

In conclusion, our results showed that acerola juice prevents weight gain (measured in terms of body weight and the adiposity index) and dyslipidemia (measured using TAG levels) and restores metabolic and inflammatory pathways to a normal range. Future studies are needed to better understand the mechanisms involved in the beneficial effects associated with acerola juice intake, especially in mice fed a cafeteria high-fat diet.

## Abbreviations

STA: Standard diet; CAF: Cafeteria diet; CAF + WD: Cafeteria diet + water distilled; CAF + IND: Cafeteria diet + acerola industrial; CAF + UNR: Cafeteria diet + acerola Unripe; CAF + RIP: Cafeteria diet + acerola Ripe; CAF + Vit C: Cafeteria diet + Vitamin C; TAG: Triglyceride; HSL: Hormone sensitive lipase; FAS: Fatty acid synthase; ACC: Acetyl CoA Carboxylase; TLR-4: Toll-like receptor 4; NFκB: Nuclear factor kappa B; IL-6: Interleukin 6; IL-10: Interleukin 10; TNF-α: Tumor necrosis factor alpha; CGI-58: Comparative gene identification 58; ATGL: Adipose triglyceride lipase; IκB-α: Inhibitor of kappa B alpha; JNK: Jun N-terminal kinases; AMPK: 5′ AMP-activated protein kinase.

## Competing interest

All authors declare no conflicts of interest.

## Authors’ contributions

FMD, DDL, FD, VMA, contributed to the study design and protocol; SOM, TFL, JCP, AAS and RXN analyzed the samples; and JCR, LMO, CTS, and FSL wrote the manuscript and conducted the statistical analysis, with input and advice from all authors. All authors read and approved the final manuscript.
